# High Correlation of 2,2-diphenyl-1-picrylhydrazyl (DPPH) Radical Scavenging, Ferric Reducing Activity Potential and Total Phenolics Content Indicates Redundancy in Use of All Three Assays to Screen for Antioxidant Activity of Extracts of Plants from the Malaysian Rainforest

**DOI:** 10.3390/antiox2010001

**Published:** 2013-01-04

**Authors:** Garry Clarke, Kang Nee Ting, Christophe Wiart, Jeffrey Fry

**Affiliations:** 1 School of Biomedical Sciences, University of Nottingham, Nottingham NG7 2UH, UK; E-Mail: garry.clarke@nottingham.ac.uk; 2 School of Biomedical Sciences, University of Nottingham, Jalan Broga, 43500, Semenyih, Selangor, Malaysia; E-Mails: Kang-Nee.Ting@nottingham.edu.my (K.N.T.); Christophe.Wiart@nottingham.edu.my (C.W.)

**Keywords:** antioxidant, plant extracts, DPPH, FRAP, phenolics, correlation

## Abstract

Extracts of plants from the Malaysian rainforest and other fragile habitats are being researched intensively for identification of beneficial biological actions, with assessment of antioxidant behavior being a common component of such assessments. A number of tests for antioxidant behavior are used, with the 2,2-diphenyl-1-picrylhydrazyl (DPPH) and ferric reduction activity potential (FRAP) assays often being used in parallel, and also with measurement of total phenolics content (TPC) as a surrogate marker for antioxidant capacity. The present study investigated the possible redundancy in using all three assays to determine antioxidant capacity in 92 extracts obtained from 27 plants from the Malaysian rainforest. The results demonstrated that the assays displayed a high (*R* ≥ 0.82) and significant (*P* < 0.0001) correlation with one another, indicating a high level of redundancy if all three assays are used in parallel. This appears to be a waste of potentially valuable plant extracts. Because of problems with the FRAP assay relating to color interference and variable rates of reaction point, the DPPH assay is the preferred assay in preliminary screening of extracts of plants from the Malaysian rainforest.

## 1. Introduction

There is considerable interest in charting the biological activities of plants from geographically remote and fragile habitats such as the primary rainforests of Malaysia and other locations in South-East Asia, in the hope of identifying novel compounds of potential therapeutic value [1].

Oxidative stress is believed to be a major contributor to the pathogenesis of a number of chronic diseases [2], and it is for this reason that antioxidant behavior is one of the most commonly determined biological activities in extracts of plants [3]. A wide variety of antioxidant assays are used when determining the antioxidant activity of plant extracts, two common ones being based on the scavenging of the DPPH (2,2-diphenyl-1-picrylhydrazyl) radical (DPPH assay) and ferric reduction activity potential (FRAP assay). Many utilize both the DPPH and the FRAP assays in their plant activity screening programs, presumably on the assumption that a combination of the data would provide a better description of antioxidant activity than obtained from a single assay (for recent examples see [4,5,6,7]). However, should this assumption not be correct, use of these two assays will use up potentially valuable plant material without providing additional information.

As part of a program mapping the biological activities of plants of the Malaysian rainforest, a total of 96 extracts isolated from 27 plants have been tested using both the DPPH and FRAP assays, and an assessment made of the correlation between the two assays. These findings are presented in this paper, together with data on the total phenolics content (TPC) of the extracts, which is often taken as an indirect measure of antioxidant capacity [8].

## 2. Experimental Section

### 2.1. Collection of Plants and Preparation of Extracts

Plant extracts were collected from various sites in the Malaysian primary rainforest, with relevant authority as required under the framework of the United Nations Convention on Biodiversity, and water (W), ethanol (EtOH), ethyl acetate (EtAc) and hexane (Hex) fractions were prepared from leaf, bark or the whole aerial parts of the plant using standard techniques [9]. A full listing of the plants and extracts is given in Table 1.

**Table 1:** Plant extracts used in this study and their antioxidant activity as determined by results of the 2,2-diphenyl-1-picrylhydrazyl (DPPH), ferric reduction activity potential (FRAP) and total phenolics content (TPC) assays.

Extract No.	Plant	Part	Extract	DPPH ^1^	FRAP ^1^	TPC ^2^
1	*Apama tricornutum*	Leaf	EtOH	2740	1617	59.6
2			EtAc	≥4000	≥4000	37.2
3			Hex	≥4000	≥4000	9.0
4		Bark	EtOH	≥4000	1842	58.0
5			EtAc	≥4000	2394	33.8
6			Hex	≥4000	≥4000	11.5
7	*Dysoxylum dumosum* King	Leaf	EtOH	476	337	182.7
8			EtAc	≥4000	≥4000	5.0
9		Bark	EtOH	556	580	134.1
10			EtAc	≥4000	≥4000	40.3
11			Hex	≥4000	≥4000	7.7
12	*Alternanthera sessilis*	Whole	EtOH	≥4000	≥4000	41.9
13			EtAc	≥4000	≥4000	33.1
14			Hex	≥4000	≥4000	7.4
15	*Borreria latifolia*	Whole	EtOH	≥4000	≥4000	37.8
16			EtAc	≥4000	≥4000	37.4
17			Hex	≥4000	≥4000	8.8
18	*Eclipta alba*	Whole	EtOH	3699	≥4000	37.7
19			EtAc	≥4000	≥4000	33.9
20			Hex	≥4000	≥4000	10.7
21	*Calotropis gigantea*	Whole	EtOH	≥4000	≥4000	35.5
22			EtAc	3674	1430	72.7
23			Hex	≥4000	≥4000	2.4
24	*Acalypha wilkesiana*	Whole	EtOH	52.2	270	421.5
25			EtAc	47.6	310	474.6
26			Hex	2170	≥4000	12.5
27	*Borreria articularis*	Whole	EtOH	≥4000	2562	39.4
28			EtAc	≥4000	3288	33.9
29			Hex	≥4000	≥4000	3.7
30	*Euphorbia hirta*	Whole	EtOH	≥4000	3918	39.2
31			EtAc	≥4000	≥4000	19.6
32			Hex	≥4000	≥4000	13.1
33	*Gluta wallichii*	Whole	EtOH	736	1110	134.6
34			EtAc	≥4000	≥4000	47.0
35			Hex	≥4000	≥4000	25.3
36	*Derris dalbergioides*	Whole	EtOH	115	339	202.0
37			EtAc	893	2293	66.8
38			Hex	≥4000	≥4000	17.3
39	*Gnetum gnemon*	Whole	EtOH	3315	2731	108.3
40			EtAc	3787	2783	185.9
41			Hex	≥4000	≥4000	9.2
42	*Polyalthia hypoleuca*	Whole	EtOH	661	1546	70.7
43			EtAc	2609	2774	53.2
44			Hex	≥4000	≥4000	19.0
45	*Sida acuta*	Whole	EtOH	531	800	113.1
46			EtAc	1897	2600	67.6
47			Hex	≥4000	≥4000	17.8
48	*Pseudo-uvaria macrophylla* (Oliv.) Merr.	Whole	EtOH	431	1260	91.9
49			EtAc	≥4000	≥4000	30.1
50			Hex	≥4000	≥4000	14.3
51	*Berberis thunbergii*	Whole	EtOH	≥4000	2553	78.5
52			EtAc	≥4000	≥4000	36.9
53			Hex	≥4000	≥4000	13.6
54	*Ipomoea quamoclit*	Whole	EtOH	3460	≥4000	24.1
55			EtAc	3210	≥4000	42.3
56	*Oldenlandia corymbosa *L.	Whole	EtOH	3740	≥4000	35.6
57			EtAc	≥4000	≥4000	15.3
58			Hex	≥4000	≥4000	8.6
59	*Bidens pilosa *L.	Whole	EtOH	720	730	83.4
60			EtAc	900	900	105.0
61	*Justicia betonica *L.	Whole	EtOH	≥4000	3040	28.6
62			EtAc	≥4000	≥4000	40.1
63			Hex	≥4000	≥4000	6.5
64	*Clerodendrum paniculatum*	Whole	EtOH	1720	2300	71.7
65			EtAc	3190	≥4000	34.5
66			Hex	≥4000	≥4000	6.1
67	*Ardisia punctata *Jack	Whole	EtOH	710	810	101.7
68			EtAc	1310	3130	83.8
69			Hex	2460	≥4000	78.0
70	*Clerodendron nutans *Jack	Whole	EtOH	1000	1040	67.1
71			EtAc	1320	1990	56.4
72			Hex	≥4000	≥4000	9.2
73	*Marsypopetallum pallidum *(Bl.) Kurz	Leaf	Water	420	710	128.3
74			EtOH	1530	1380	76.9
75			EtAc	≥4000	≥4000	19.2
76			Hex	≥4000	≥4000	16.7
77	*Archidendron ellipticum*	Leaf	EtOH	1310	1130	97.3
78			EtAc	1930	2420	60.0
79			Hex	≥4000	≥4000	8.6
80		Bark	EtOH	1970	2310	32.3
81			EtAc	≥4000	≥4000	25.0
82			Hex	≥4000	≥4000	9.8
83	*Pipturus argenteus* (Forst. f.)	Leaf	EtOH	340	940	119.2
84			EtAc	3520	≥4000	19.3
85			Hex	≥4000	≥4000	12.0
86		Bark	EtOH	540	550	174.6
87			EtAc	≥4000	≥4000	28.7
88			Hex	≥4000	≥4000	6.4
89	*Duabanga grandiflora*	Leaf	Water	140	210	251.9
90			EtOH	50.0	110	476.8
91			EtAc	150	280	192.3
92			Hex	≥4000	≥4000	10.4
93		Bark	Water	130	260	363.7
94			EtOH	80.0	210	404.6
95			EtAc	240	570	135.4
96			Hex	≥4000	≥4000	18.9
	Quercetin			59.8 ± 0.75 ^3^	49.9 ± 1.02 ^3^	N/A

^1^ Activity measured as EC_50_ (μg/mL) for DPPH assay or FE (μg/mL) for FRAP assay.

^2^ Content measured as μg quercetin equivalents per mg of extract.

^3^ Mean ± SEM (*n* = 5–6).

### 2.2. Chemicals

DPPH, DMSO, quercetin, tripyridyltriazine (TPTZ) and Folin-Ciocalteau (F-C) reagent were obtained from Sigma-Aldrich, Dorset, UK. All other chemicals were of the highest analytical grade available from local suppliers.

### 2.3. DPPH Assay

For the DPPH radical scavenging assay, 20 μL of extract diluted appropriately in DMSO was mixed with 180 μL of DPPH in methanol (40 μg/mL) in wells of a 96-well plate. The plate was kept in the dark for 15 min, after which the absorbance of the solution was measured at 540 nm in a Multiskan Ascent plate-reader (Thermo Electron Corporation, Basingstoke, UK). Appropriate blanks (DMSO) and standards (quercetin solutions in DMSO) were run simultaneously. Extracts were first tested at a single concentration of 4 mg/mL, and those showing good evidence of antioxidant activity were tested over a range of concentrations to establish the EC_50_ (the concentration reducing DPPH absorbance by 50%). This method follows closely that used by previous workers [4,5,6,7].

### 2.4. FRAP Assay

For determination of FRAP response, 20 μL of extract diluted appropriately in DMSO was mixed with 180 μL FRAP reagent in wells of a 96-well plate, left for 6 minutes, and the absorbance measured at 595 nm in a Multiskan Ascent plate-reader. FRAP reagent was prepared freshly by mixing 300 mM acetate buffer pH 3.6, 10 mM TPTZ in 40 mM HCl, and 20 mM FeCl_3_.6H_2_0 in the volume ratio 10:1:1. Appropriate blanks of plant extract and of FRAP reagent lacking TPTZ (to correct for color of the extracts) were run, together with quercetin (in DMSO) and FeSO_4_ as a standard. FRAP activity was calculated as Ferrous Equivalents (FE), the concentration of extract/quercetin which produced an absorbance value equal to that of 1 mM FeSO_4_. Once again, extracts were first tested at a single concentration of 4 mg/mL, and those showing good evidence of antioxidant activity were tested over a range of concentrations to establish the FE. This method follows closely that used by previous workers [4,5,6,7].

### 2.5. TPC Assay

The total phenolics content of the extracts was determined by reaction with F-C reagent. Thus, 10 μL of extract diluted appropriately in DMSO was mixed with 100 μL F-C reagent freshly diluted 1/10 with distilled water. After five minutes, the solution was mixed with 100 μL 7.5% Na_2_CO_3_ solution, and the whole left for 60 min, before measurement of absorbance at 650 nm in a Multiskan Ascent plate-reader. Appropriate blanks (DMSO) and standard (quercetin in DMSO) were run simultaneously, after which the total phenolics content was calculated as μg quercetin equivalents per mg extract. This method follows closely that used by previous workers [4,5,6,7].

### 2.6. Data Analysis

Correlation and regression analysis of the data were performed using the GraphPad Prism v4 program.

## 3. Results and Discussion

### 3.1. Results

**Figure 1:**
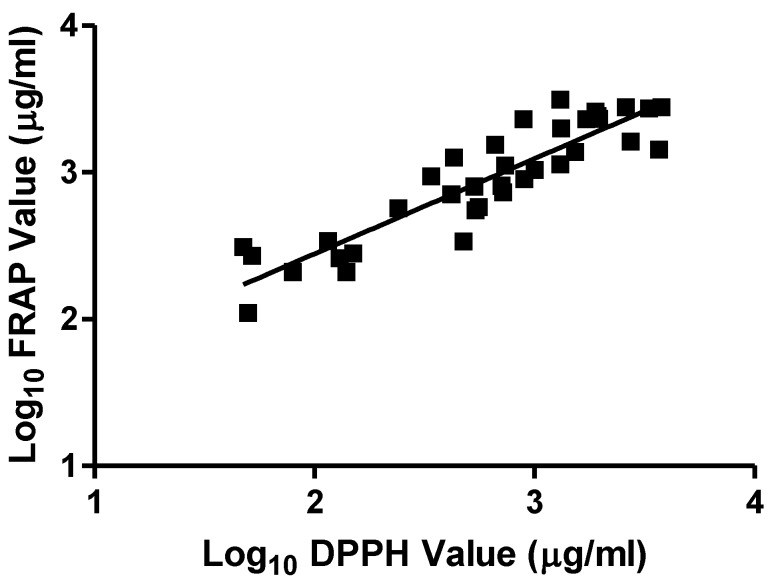
Correlation of DPPH value (as EC_50_ in μg/mL) and FRAP value (as FE in μg/mL) for the 36 plant extracts indicated in Table 1 with EC_50_ and FE values of less than 4000 μg/mL, presented on log_10_ scales.

The results from the DPPH, FRAP, and TPC assays are presented in Table 1. Of the 96 extracts tested, many were inactive in both of the antioxidant assays (EC_50_ or FE greater than 4000 μg/mL). Nevertheless, non-parametric Spearman correlation analysis of all 96 data points demonstrated highly significant (*P* < 0.0001) positive correlation between the DPPH and FRAP results (*R* = 0.852). When the extracts for which both DPPH and FRAP values were greater than 4000 μg/mL were excluded, the remaining 36 extracts still displayed a highly significant correlation (Spearman *R* = 0.889). This is exemplified in Figure 1, in which the log_10_ values for these 36 extracts are plotted, linear regression yielding the following equation:

Log_10_ FRAP = 0.6484 Log_10_ DPPH + 0.8440 (*R*^2^ = 0.8162; *P* < 0.0001) (1)

**Figure 2:**
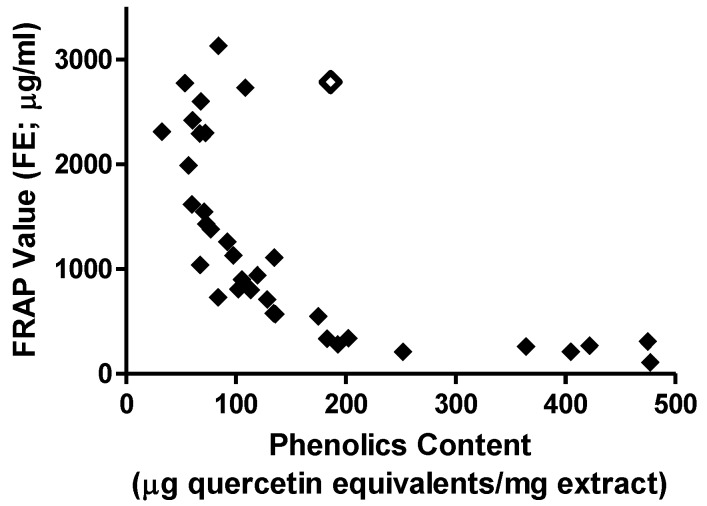
Relation between FRAP value (as FE in μg/mL) and TPC (as μg quercetin equivalents/mg extract) for the 36 plant extracts indicated in Table 1 with FE value less than 4000 μg/mL.

There was also a highly significant (*P* < 0.0001) negative correlation between the DPPH/FRAP and TPC values for all 96 extracts (Spearman *R* for DPPH = −0.821 and for FRAP = −0.848). In general, those extracts for which the results of one or both of the DPPH and FRAP assays yielded EC_50_/FE values greater than 4000 μg/mL possessed a TPC value of approximately 55 μg quercetin equivalents per mg extract or less. For those extracts displaying EC_50_/FE values less than 4000 μg/mL, a curvilinear relationship of DPPH/FRAP against TPC was apparent (see Figure 2 for FRAP data), with extracts displaying a TPC in excess of 200 μg quercetin equivalents per mg extract also displaying high antioxidant activity by either DPPH or FRAP assay. One possible outlier is extract 40 (open diamond symbol in Figure 2); the reason for this remains to be determined.

**Figure 3:**
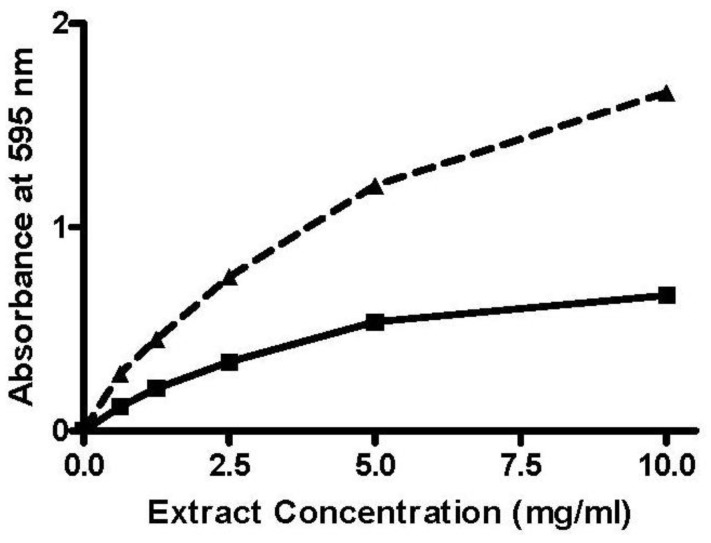
Concentration dependence for FRAP reaction of plant extract 2, measuredafter incubation for 6 min (▄) or 3 h (▲).

Two methodological problems became apparent during the early stages of performing the FRAP assays. The first problem to emerge was that the color of some of the extracts interfered in the assay, and “color controls” (FRAP reagent in which the TPTZ solution was replaced with an equal volume of 40 mM HCl) had to be introduced; this did not appear to be a major issue with the DPPH assay. The second problem to emerge was that, for some extracts, the color reaction was not complete within the 6-min assay period; an example is shown in Figure 3.

### 3.2. Discussion

The results of this investigation have demonstrated that screening for antioxidant activity of extracts of plants from the Malaysian rainforest by DPPH and FRAP assays gives essentially identical results. In addition, it was apparent that extracts displaying good antioxidant behavior in both the DPPH and FRAP assays could be identified by high phenolics content, and that methodological issues with the FRAP assay may potentially compromise the usefulness of this assay when testing plant extracts.

The finding that the results of the DPPH and FRAP assays for plant extracts were highly correlated agrees with the work of others (for examples, see [4,10,11]), and is consistent with the view that the two assays share a similar mechanistic basis, *viz*. transfer of electrons from the antioxidant to reduce an oxidant, as proposed by Huang, Ou and Prior [8]. A number of papers have reported results from both DPPH and FRAP (or other Fe^3+^-reduction) assays on plant extracts (see [4,5,6,7]), presumably in the belief that using two assays improves the overall estimate of antioxidant capacity of the plant extracts, and/or that each assay reflects a different aspect of the antioxidant behavior of a plant extract. However, the results from the present and previous studies, together with the similar mechanistic basis of the assays, suggest a high degree of redundancy in use of both assays for screening plant extracts.

Under these circumstances, a decision needs to be made as to which assay to use to screen for antioxidant activity, so as to reduce the use of potentially valuable plant extracts. In the work reported here, two problems were identified with the use of the FRAP assay—interference caused by the color in some extracts, and slow development of color. The former was possibly due to the acid pH under which the FRAP assay was run, and was much less of a problem with the DPPH assay. The slow development of color has been reported in other studies [12,13,14], and has been taken to indicate the involvement of multiple antioxidants in the observed response, each acting under different kinetic conditions. Although similar issues have been reported for the DPPH assay [13,14], this did not appear to be a major problem in the studies reported here or in subsequent studies with other plant extracts [15].

The TPC assay is another assay that is commonly used in conjunction with either or both of the DPPH and FRAP assays, again presumably with the aim of increasing the information database on a particular plant extract. The results presented in this study indicate that high antioxidant activity is associated with a high phenolics content, a finding reported previously many times (for recent examples see [4,6,10,16]), so it could be argued that the only virtue in performing the TPC assay would be as a screen to evaluate extracts further by either the DPPH or FRAP assays. Under such circumstances, the critical point would then become the threshold value above which further screening would be undertaken. This value will obviously depend upon the standard being used for the TPC assay, but under the present experimental conditions, this threshold value would appear to be 200 μg quercetin equivalents per mg extract. 

It should be noted that the assays used in this work represent only a few of the many antioxidant assays available (see review by Huang, Ou and Prior [8]), albeit three of the most popular in studies of antioxidant capacity of plant extracts, as exemplified by examples referenced above.

## 4. Conclusions

In conclusion, this study has indicated that results of the DPPH, FRAP and TPC assays provide essentially identical information in regard to the antioxidant capability of extracts of plants from the Malaysian rainforest, so that it is difficult to establish what additional information could be gained by use of more than one of the three assays employed in this study. Given this level of redundancy with these assays, two screening scenarios can be proposed—screening of all extracts by DPPH assay or determination of TPC followed by more detailed analysis by DPPH assay for those extracts demonstrating a total phenolics content of 200 μg quercetin equivalents per mg extract or more. The former approach is currently being used in characterization of other extracts of plants from the Malaysian rainforest. 
